# Clinical Outcomes and Acute Exacerbation Incidence in Combined Pulmonary Fibrosis and Emphysema

**DOI:** 10.1002/resp.70285

**Published:** 2026-07-08

**Authors:** Kazuya Tsubouchi, Yasuaki Kotetsu, Tomotsugu Takano, Hiroaki Ogata, Fumiaki Kiyomi, Katsuyuki Ichiki, Shohei Takata, Yasuhiko Kitasato, Hiroshi Ishii, Kazuhiro Yatera, Kei Yamasaki, Masaki Okamoto, Makoto Yoshida, Masayuki Kawasaki, Masaki Fujita, Tomohiro Ogawa, Kazunori Tobino, Eiji Harada, Noriaki Nakagaki, Masashi Komori, Taishi Harada, Hiroshi Wataya, Yoichi Nakanishi, Isamu Okamoto

**Affiliations:** ^1^ Department of Respiratory Medicine Graduate School of Medical Sciences, Kyushu University Fukuoka Japan; ^2^ Clinical Research Support Center Kyushu Fukuoka Japan; ^3^ Kirigaoka Tsuda Hospital Kitakyushu Japan; ^4^ Department of Respiratory Diseases NHO Fukuokahigashi Medical Center Koga Japan; ^5^ Department of Respiratory Medicine Japan Community Health Care Organization Kurume General Hospital Kurume Japan; ^6^ Department of Respiratory Medicine Fukuoka University Chikushi Hospital Chikushino Japan; ^7^ Department of Respiratory Medicine School of Medicine, University of Occupational and Environmental Health, Japan Kitakyushu Japan; ^8^ Department of Respiratory Medicine Wakamatsu Hospital of the University of Occupational and Environmental Health, Japan Kitakyushu Japan; ^9^ Division of Respirology, Neurology, and Rheumatology, Department of Internal Medicine Kurume University School of Medicine Kurume Japan; ^10^ Department of Respiratory Medicine NHO Fukuoka National Hospital Fukuoka Japan; ^11^ Department of Respiratory Diseases NHO Omuta National Hospital Omuta Japan; ^12^ Department of Respiratory Medicine Fukuoka University School of Medicine Fukuoka Japan; ^13^ Department of Respiratory Medicine Hamanomachi Hospital Fukuoka Japan; ^14^ Division of Respiratory Medicine Aso Iizuka Hospital Iizuka Japan; ^15^ Department of Respiratory Medicine Kitakyushu Municipal Medical Center Kitakyushu Japan; ^16^ Department of Respiratory Medicine Japanese Red Cross Fukuoka Hospital Fukuoka Japan; ^17^ Department of Respiratory Medicine Steel Memorial Yawata Hospital Kitakyushu Japan; ^18^ Department of Respiratory Medicine Japan Community Health Care Organization Kyushu Hospital Kitakyushu Japan; ^19^ Department of Respiratory Medicine Harasanshin Hospital Fukuoka Japan; ^20^ Kitakyushu City Hospital Organization Kitakyushu Japan

**Keywords:** acute exacerbation, CPFE, emphysema, pulmonary fibrosis, visual assessment

## Abstract

**Background and Objective:**

Combined pulmonary fibrosis and emphysema (CPFE) is characterized by coexisting upper‐zone emphysema and lower‐zone fibrosis, but standardized diagnostic criteria and clinical outcomes remain poorly defined. This study aimed to evaluate survival, acute exacerbation incidence, and disease progression in CPFE patients using international classification criteria with visual assessment of emphysema extent.

**Methods:**

This multicentre prospective cohort study at 29 Japanese hospitals enrolled 1016 patients with idiopathic interstitial pneumonias (IIPs) or chronic obstructive pulmonary disease (COPD) between 2013 and 2016. CPFE was defined as ≥ 5% emphysema determined by visual assessment of CT scans for IIP patients, according to international classification criteria. Patients were followed for 5 years.

**Results:**

Among 528 IIP patients, 92 (17.4%) had CPFE. CPFE patients showed a significantly worse 5‐year survival compared with non‐CPFE IIP patients (51.4% vs. 63.1%, *p* = 0.026) and COPD patients (51.4% vs. 84.0%, *p* < 0.001). After adjustment for covariates including IIP subtype, CPFE remained independently associated with increased mortality among IIP patients (HR of 1.85 [95% CI, 1.27–2.69], *p* < 0.001). CPFE patients also had a significantly higher acute exacerbation rate than did non‐CPFE IIP patients (*p* < 0.001). The prognostic impact of CPFE was most pronounced in patients with idiopathic pulmonary fibrosis (IPF), with CPFE/IPF patients showing the worst outcomes.

**Conclusion:**

Visual assessment with a ≥ 5% emphysema threshold effectively identified CPFE patients with a distinct clinical phenotype characterized by poor survival and an increased acute exacerbation incidence. These findings validate international classification criteria and emphasize the need for enhanced surveillance and tailored management strategies for this high‐risk population.

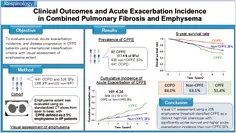

## Graphical abstract

This multicentre prospective study of 1016 patients demonstrated that CPFE, defined as ≥ 5% emphysema on visual CT assessment, identified a high‐risk phenotype with significantly worse 5‐year survival compared to non‐CPFE IIP patients (51.4% vs. 63.1%) and COPD patients (51.4% vs. 84.0%) and higher acute exacerbation incidence, validating international classification criteria.

## 1 Introduction

Combined pulmonary fibrosis and emphysema (CPFE) is a recognized clinical syndrome characterized by the coexistence of upper‐zone emphysema and lower‐zone pulmonary fibrosis as detected by high‐resolution computed tomography (CT) [1]. CPFE mostly affects male smokers and presents with preserved lung volumes as a result of the opposing effects of emphysema and fibrosis, whereas diffusion capacity is markedly reduced [2]. Although multiple studies have described various clinical characteristics of CPFE, the interpretation of such findings has been hampered by substantial heterogeneity in classification criteria [1, 3, 4, 5, 6, 7]. Previous studies have adopted markedly different emphysema thresholds ranging from > 0% to > 15%, making it impossible to establish consistent clinical outcomes or to develop standardized management approaches and hindering the formulation of evidence‐based therapeutic guidelines.

This classification heterogeneity was addressed by the landmark 2022 ATS/ERS/JRS/ALAT research statement, which provided the first international consensus on CPFE definition and classification criteria [8]. This pivotal publication recommended standardized emphysema assessment methods and thresholds, proposing a unified framework that allows meaningful comparison across studies and institutions [8, 9]. These recommendations now provide the foundation for conducting rigorous, comparable research in CPFE populations.

CPFE has several distinctive clinical characteristics, including a relatively high prevalence of pulmonary hypertension and increased lung cancer risk, contributing to poor prognosis [6, 8, 10, 11]. However, acute exacerbation, another key prognostic event in interstitial lung diseases (ILDs), remains poorly characterized in CPFE populations, with limited data available in the literature [12, 13, 14, 15, 16, 17, 18]. In addition, whereas the current international consensus does not specify the underlying ILD type in the definition of CPFE, prognosis may vary substantially depending on whether it occurs with idiopathic pulmonary fibrosis (IPF), non‐IPF idiopathic interstitial pneumonias (IIPs), connective tissue disease–related lung disease, or occupational and environmental ILDs, given that these underlying conditions have distinct pathophysiologies and disease behaviours [8, 19, 20, 21].

We have therefore conducted a large multicentre prospective cohort study to validate the international CPFE classification criteria and to evaluate clinical outcomes, including survival and acute exacerbation rates, across different IIP subtypes.

## 2 Methods

### 2.1 Study Subjects

This prospective, multicentre observational study was conducted by the Fukuoka Tobacco‐Related Lung Disease (FOLD) registry study group across 29 general hospitals in Fukuoka, Japan [22]. The main inclusion criterion was age > 20 years, and both newly and previously diagnosed patients were sequentially enrolled after a diagnosis of IIPs or chronic obstructive pulmonary disease (COPD) was made by respiratory specialists at each participating facility. A total of 1016 patients diagnosed with IIPs or COPD were enrolled between September 2013 and April 2016. Patients with active malignancy at enrollment were excluded to avoid confounding effects on survival outcomes. All diagnoses were reassessed by a central diagnosis committee consisting of pulmonologists, subspecialist radiologists, and histopathologists. The diagnosis of COPD was made by clinicians based on the symptoms and clinical data including spirometry according to international guidelines [23], and IIP subtypes were classified according to the ATS/ERS/JRS/ALAT guidelines through multidisciplinary discussion [24, 25].

### 2.2 Visual Assessment of Emphysema

Visual assessment of emphysema extent was performed for all enrolled patients with the use of six representative CT slices from apex to base according to established methods referenced in the 2022 ATS/ERS/JRS/ALAT research statement [9]. Six anatomically defined axial sections were selected with the use of standardized landmarks: the first at the aortic arch level, the second 1 cm below the carina, the third at the pulmonary venous confluence, the fourth equidistant between the third and fifth sections, the fifth 2 cm above the right hemidiaphragm, and the sixth 1 cm below the right hemidiaphragm. This approach emphasized lower lung zones to ensure adequate assessment of fibrotic changes, which is essential for CPFE diagnosis. Emphysema extent was independently evaluated by three experienced pulmonologists, and the final assessment was determined by averaging their scores. CPFE was defined as the presence of emphysema of ≥ 5% by visual assessment in patients with IIPs. The interobserver agreement among the three pulmonologists for visual assessment of emphysema extent was excellent, with an intraclass correlation coefficient (ICC) of 0.886. Representative CT images illustrating emphysema extents of approximately 5%, 10%, 20%, and 30% are provided in Figure S1 to aid visual interpretation of the assessment criteria.

### 2.3 Data Collection

Annual follow‐up surveillance was conducted for 5 years to evaluate disease progression, acute exacerbations, and death. Laboratory tests including KL‐6 (Krebs von den Lungen‐6), a representative biomarker for IIPs, were performed. For patients with IIPs, acute exacerbation was defined by all of the following criteria being met within a period of 1 month after ruling out alternative diagnoses such as pulmonary infection, pneumothorax, malignancy, pulmonary embolism, or heart failure: (i) worsening dyspnoea, (ii) new bilateral ground‐glass opacities and/or consolidation superimposed on a background of reticular or honeycomb patterns, and (iii) a decrease in PaO_2_ of > 10 mmHg from baseline [26].

### 2.4 Statistical Analysis

Continuous variables are presented as median and interquartile range, categorical variables as frequencies with percentages. Between‐group comparisons used chi‐square or Fisher's exact test for categorical variables and Wilcoxon rank‐sum test for ordered categorical and continuous variables. Kaplan–Meier survival analysis estimated overall survival and cumulative incidence of acute exacerbations, with log‐rank test for comparisons. Cox proportional hazards regression calculated hazard ratios (HR) and 95% confidence intervals (CI), adjusted for age, sex, percent predicted forced vital capacity (FVC), smoking pack‐years, and IIP subtype where appropriate. Statistical analyses used SAS 9.4 (SAS Institute). Two‐sided *p* < 0.05 was considered statistically significant.

## 3 Results

### 3.1 Study Subjects

Between September 2013 and April 2016, a total of 1024 patients were recruited. Eight individuals were subsequently excluded, with four declining participation, two with missing test results, one who failed to meet the inclusion criteria, and one being excluded for unknown reasons. The remaining 1016 patients were enrolled sequentially after a diagnosis of either IIP or COPD. All diagnoses of the enrolled patients were reevaluated through a multidisciplinary discussion among pulmonologists, radiologists, and histopathologists, with the result that 543 patients were diagnosed with IIPs and 461 with COPD. Patients lost to follow‐up included 15 with IIPs and 20 with COPD, leaving 528 IIP patients and 441 COPD patients for analysis in the present study. The extent of emphysema was visually assessed from chest CT images obtained at enrollment for the 528 IIP patients (306 with IPF and 222 with non‐IPF IIPs), with 92 individuals being classified as having CPFE, characterized by emphysema of ≥ 5%, and the remaining 436 patients being categorized as having non‐CPFE IIPs (Figure 1).

**FIGURE 1:**
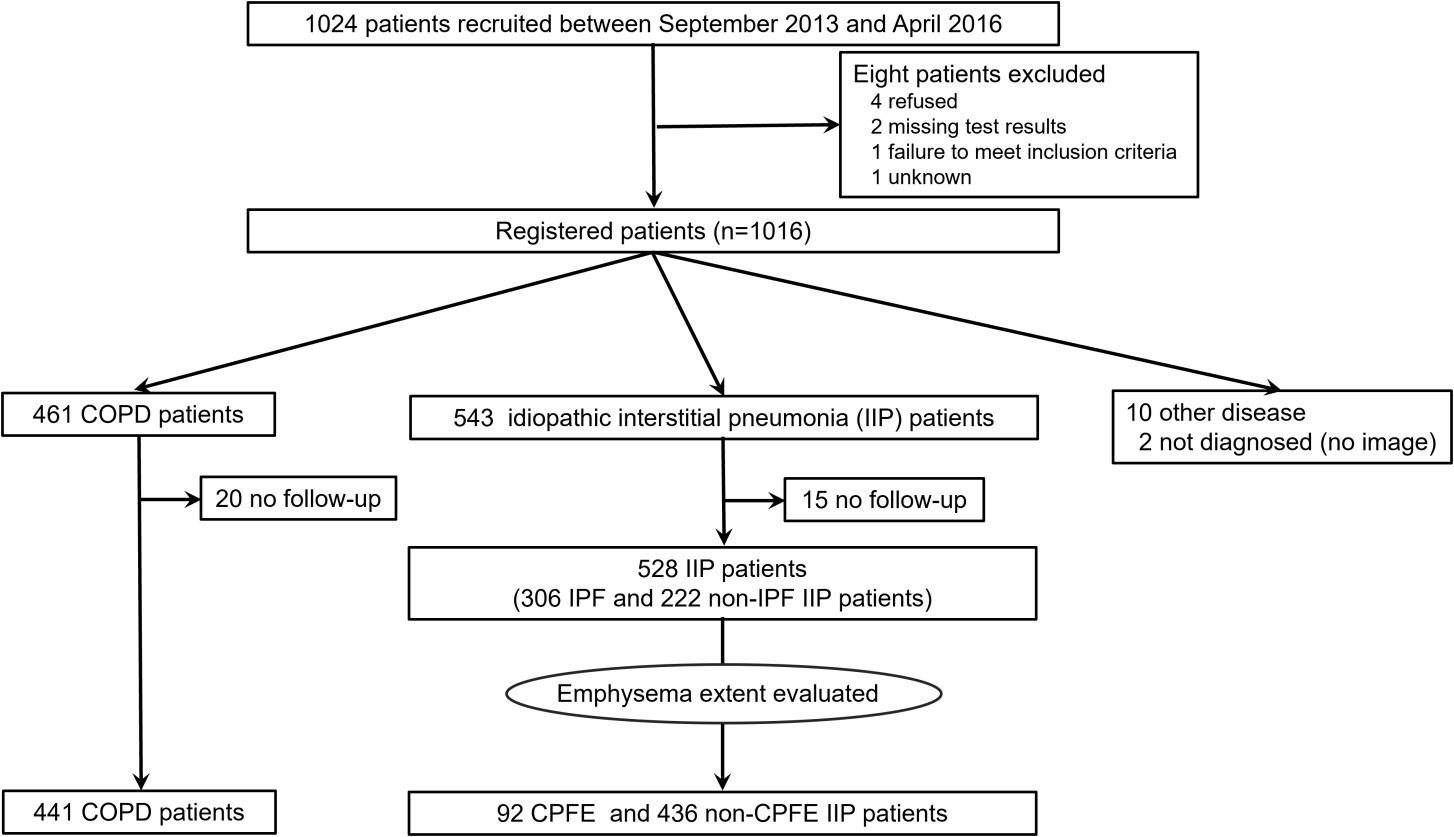
Study flow chart. COPD, chronic obstructive pulmonary disease; CPFE, combined pulmonary fibrosis and emphysema; IPF, idiopathic pulmonary fibrosis.

### 3.2 Baseline Characteristics

The clinical characteristics of patients with CPFE, non‐CPFE IIPs, or COPD are shown in Table 1. The median age was 73 years for both CPFE and non‐CPFE IIP groups and 72 years for COPD patients. The proportion of men was highest in the CPFE group. A markedly higher proportion of patients had a history of smoking in the CPFE (95.7%) and COPD (97.5%) groups compared with the non‐CPFE IIP group (64.9%). A history of lung cancer was also more common in the CPFE group (6.5%) compared with the non‐CPFE IIP group (0.7%) as well as the COPD group (2.5%). Body mass index was highest in non‐CPFE IIP patients, followed by those with CPFE, with the lowest values being apparent in the COPD group (*p* < 0.001).

**TABLE 1:** Baseline characteristics of patients with combined pulmonary fibrosis and emphysema (CPFE), non‐CPFE idiopathic interstitial pneumonias (IIPs), or chronic obstructive pulmonary disease (COPD) at registration.

Characteristic	Non‐CPFE IIPs (*n* = 436)	CPFE (*n* = 92)	COPD (*n* = 441)	*p* value among all three groups	*p* value for non‐CPFE IIPs vs. CPFE	*p* value for COPD vs. CPFE
Age (years)	73 (67–78)	73 (69–79)	72 (66–78)	0.372	0.264	0.158
Sex						
Male	303 (69.5)	87 (94.6)	385 (87.3)	< 0.001	< 0.001	0.047
Female	133 (30.5)	5 (5.4)	56 (12.7)			
Smoking (pack‐years)	20.0 (0.0–43.0)	50.0 (35.0–76.5)	50.0 (37.5–75.0)	< 0.001	< 0.001	0.927
Never	153 (35.1)	4 (4.3)	11 (2.5)	< 0.001	< 0.001	0.018
Former	236 (54.1)	78 (84.8)	325 (73.7)			
Current	47 (10.8)	10 (10.9)	105 (23.8)			
Medical history						
Lung cancer	3 (0.7)	6 (6.5)	11 (2.5)	0.002	0.001	0.093
Other cancer	38 (8.7)	13 (14.1)	53 (12.0)	0.156	0.110	0.576
Body mass index (kg/m^2^)	23.3 (21.1–25.6)	22.7 (20.8–24.2)	21.9 (19.2–24.1)	< 0.001	0.034	0.049
FVC (L)	2.43 (1.90–3.00)	2.96 (2.53–3.38)	2.75 (2.08–3.33)	< 0.001	< 0.001	0.011
FVC (% predicted)	81.0 (67.8–96.2)	91.9 (77.0–106.3)	85.6 (69.5–101.3)	< 0.001	< 0.001	0.01
FEV_1_ (L)	2.00 (1.54–2.38)	2.17 (1.64–2.58)	1.40 (0.93–1.93)	< 0.001	0.179	< 0.001
FEV_1_ (% predicted)	81.9 (72.1–94.5)	79.4 (62.8–96.6)	54.6 (39.4–69.1)	< 0.001	0.092	< 0.001
FEV_1_/FVC (%)	82.6 (76.9–88.4)	70.9 (60.5–79.6)	53.0 (40.7–64.3)	< 0.001	< 0.001	< 0.001
DLCO (% predicted)	64.5 (51.8–81.2)	49.8 (37.0–65.8)	78.9 (59.6–98.9)	< 0.001	0.014	< 0.001
IIP subtype						
IPF	263 (60.3)	43 (46.7)			0.017	
Non‐IPF	173 (39.7)	49 (53.3)				
KL‐6 (U/mL)	749 (488–1198)	642 (420–978)	283 (214–398)	< 0.001	0.046	< 0.001

*Note:* Data are presented as median (interquartile range) for continuous variables and *n* (%) for categorical variables. The *p*‐values were determined with the chi‐square test or Fisher's exact test for nominal scale variables, or with the Wilcoxon rank‐sum test for ordered categorical variables and continuous variables.

Abbreviations: DLCO, diffusion capacity of the lung for carbon monoxide; FEV_1_, forced expiratory volume in 1 s; FVC, forced vital capacity; IPF, idiopathic pulmonary fibrosis; KL‐6, Krebs von den Lungen‐6.

Pulmonary function tests revealed that FVC as a percentage of the predicted value was highest in CPFE patients (*p* < 0.001), whereas the diffusion capacity of carbon monoxide (DLCO) as a percentage of the predicted value was lowest in this group (*p* < 0.001). The forced expiratory volume in 1 s (FEV_1_) was significantly lower in the COPD group than in the other two groups (*p* < 0.001). The proportion of participants diagnosed with IPF was 46.7% in the CPFE group and 60.3% in the non‐CPFE group (*p* = 0.017). Serum KL‐6 levels were highest in non‐CPFE IIP patients, and those in the CPFE group were significantly higher than those in the COPD group (*p* < 0.001).

Treatment regimens are presented in Table S1. Non‐CPFE IIP patients had higher rates of treatment with steroids, immunosuppressants, and antifibrotic agents. Conversely, bronchodilators such as long‐acting β‐agonists and long‐acting muscarinic antagonists were administered more frequently in COPD patients. Few CPFE patients received treatment for interstitial pneumonia, with patients in this group being more likely to be on bronchodilators. Home oxygen therapy was administered most frequently in the CPFE group.

### 3.3 Survival Time

During the 5‐year follow‐up of 969 patients, 40 of 92 CPFE patients, 146 of 436 non‐CPFE IIP patients, and 63 of 441 COPD patients died (Table 2). The 5‐year survival rate was 51.4% (95% CI, 39.9%–61.7%) for CPFE, 63.1% (95% CI, 58.1%–67.7%) for non‐CPFE IIPs, and 84.0% (95% CI, 80.0%–87.3%) for COPD (Figure 2). Analysis adjusted for age, sex, %FVC, and smoking pack‐years revealed that CPFE patients had a significantly shorter overall survival compared with both non‐CPFE IIP (HR of 1.51 [95% CI, 1.05–2.18], *p* = 0.026) and COPD (HR of 5.08 [95% CI, 3.38–7.62], *p* < 0.001) patients (Table S2). The definition of CPFE on the basis of a 5% threshold for emphysema determined by visual assessment thus effectively stratified patients according to survival outcomes. The application of higher emphysema thresholds of 10% or 15% for definition of CPFE showed a numerically lower survival rate, although the differences across thresholds were modest (Figure S2).

**TABLE 2:** Causes of death in patients with combined pulmonary fibrosis and emphysema (CPFE), non‐CPFE idiopathic interstitial pneumonias (IIPs), or chronic obstructive pulmonary disease (COPD).

	Non‐CPFE IIPs	CPFE	COPD	Total
Total patient number	436	92	441	969
Deaths	146 (33.5)	40 (43.5)	63 (14.3)	249 (25.7)
Cause of death				
Chronic respiratory failure	54 (37.0)	10 (25.0)	11 (17.5)	75 (30.1)
Acute exacerbation	26 (17.8)	4 (10.0)	5 (7.9)	35 (14.1)
Pneumonia	14 (9.6)	5 (12.5)	11 (17.5)	30 (12.0)
Other infectious diseases	0 (0.0)	0 (0.0)	2 (3.2)	2 (0.8)
Lung cancer	7 (4.8)	3 (7.5)	9 (14.3)	19 (7.6)
Pulmonary hypertension	4 (2.7)	2 (5.0)	0 (0.0)	6 (2.4)
Chronic heart failure	11 (7.5)	1 (2.5)	2 (3.2)	14 (5.6)
Unknown	13 (8.9)	7 (17.5)	11 (17.5)	31 (12.4)
Other	35 (24.0)	14 (35.0)	18 (28.6)	67 (26.9)

*Note:* Data are presented as *n* (%). Multiple causes of death were identified for some patients.

**FIGURE 2:**
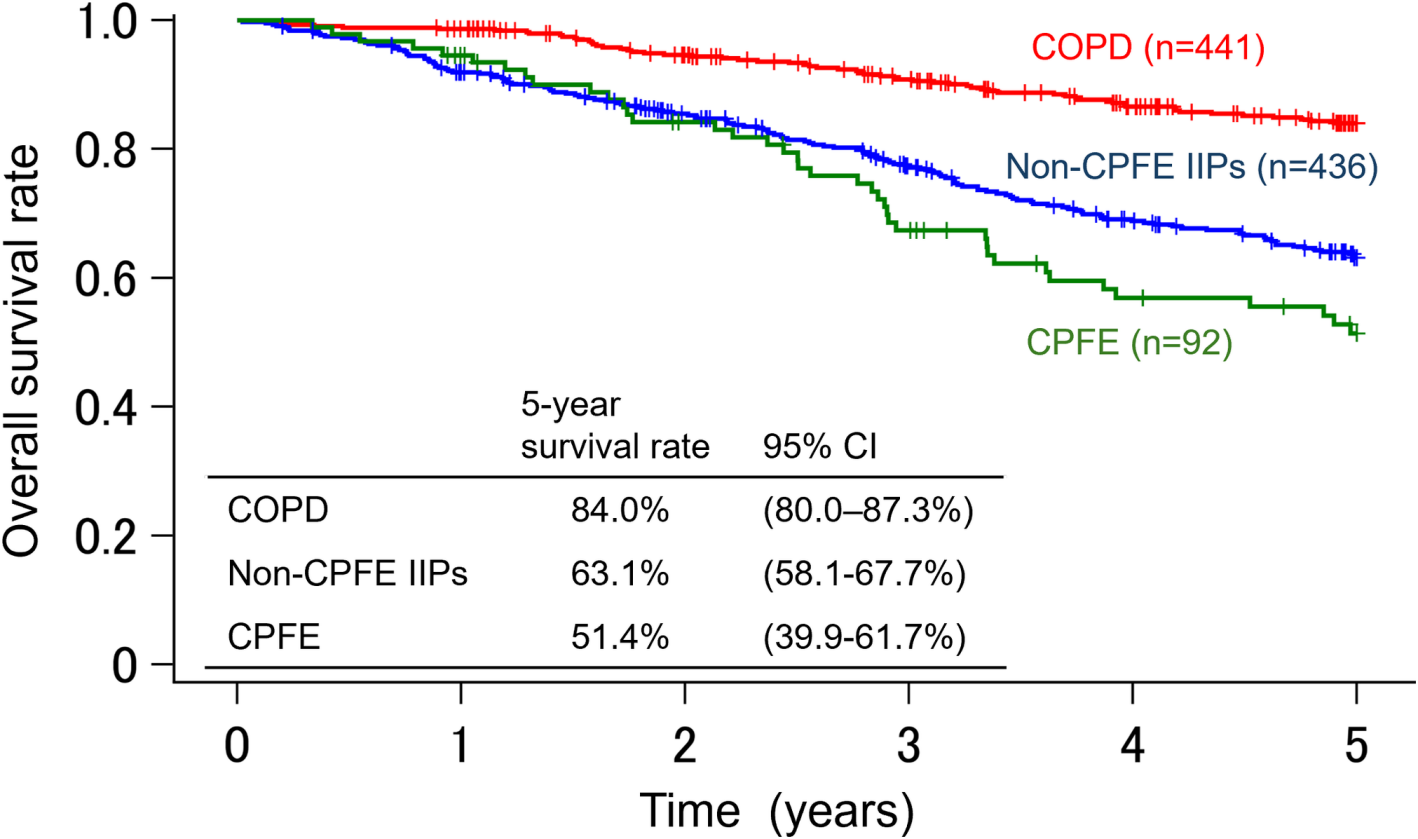
Kaplan–Meier curves of overall survival probability from the time of registration for patients with combined pulmonary fibrosis and emphysema (CPFE), non‐CPFE idiopathic interstitial pneumonias (IIPs), or chronic obstructive pulmonary disease (COPD). The number of deaths was 40 for CPFE, 146 for non‐CPFE IIP, and 63 for COPD patients. CI, confidence interval.

### 3.4 Causes of Death and Change in Lung Function

The most common cause of death across all groups was chronic respiratory failure due to the underlying disease (CPFE, 25.0%; non‐CPFE IIPs, 37.0%; COPD, 17.5%) (Table 2). Deaths attributable to pneumonia accounted for 12.5% in the CPFE group, 9.6% in the non‐CPFE IIP group, and 17.5% in the COPD group. Lung cancer was more frequently the cause of death in the COPD group (14.3%) than in the CPFE (7.5%) and non‐CPFE IIP (4.8%) groups. Deaths related to pulmonary hypertension were more common in the CPFE group (5.0%) relative to the non‐CPFE IIP group (2.7%), and they were absent in the COPD group.

Given that chronic respiratory failure was the predominant cause of death for all groups, we examined longitudinal changes in lung function parameters to characterize the patterns of disease progression. Lung function evaluations conducted annually revealed a progressive decline in predicted FVC in both the CPFE and non‐CPFE IIP groups, whereas the decline in predicted DLCO was more pronounced in the CPFE group (see Figure S3).

### 3.5 Acute Exacerbations in Patients With CPFE or Non‐CPFE IIPs

Given that acute exacerbations are key prognostic events in IIPs, we investigated the occurrence of such exacerbations in patients with CPFE or non‐CPFE IIPs. The incidence of first‐time acute exacerbations was significantly higher in CPFE patients than in the non‐CPFE patients (*p* < 0.001) (Figure 3). Multivariable analysis adjusted for age, sex, %FVC, and smoking pack‐years revealed that CPFE patients had a significantly higher risk of acute exacerbations compared with non‐CPFE IIP patients (HR of 4.34 [95% CI, 2.79–6.77], *p* < 0.001).

**FIGURE 3:**
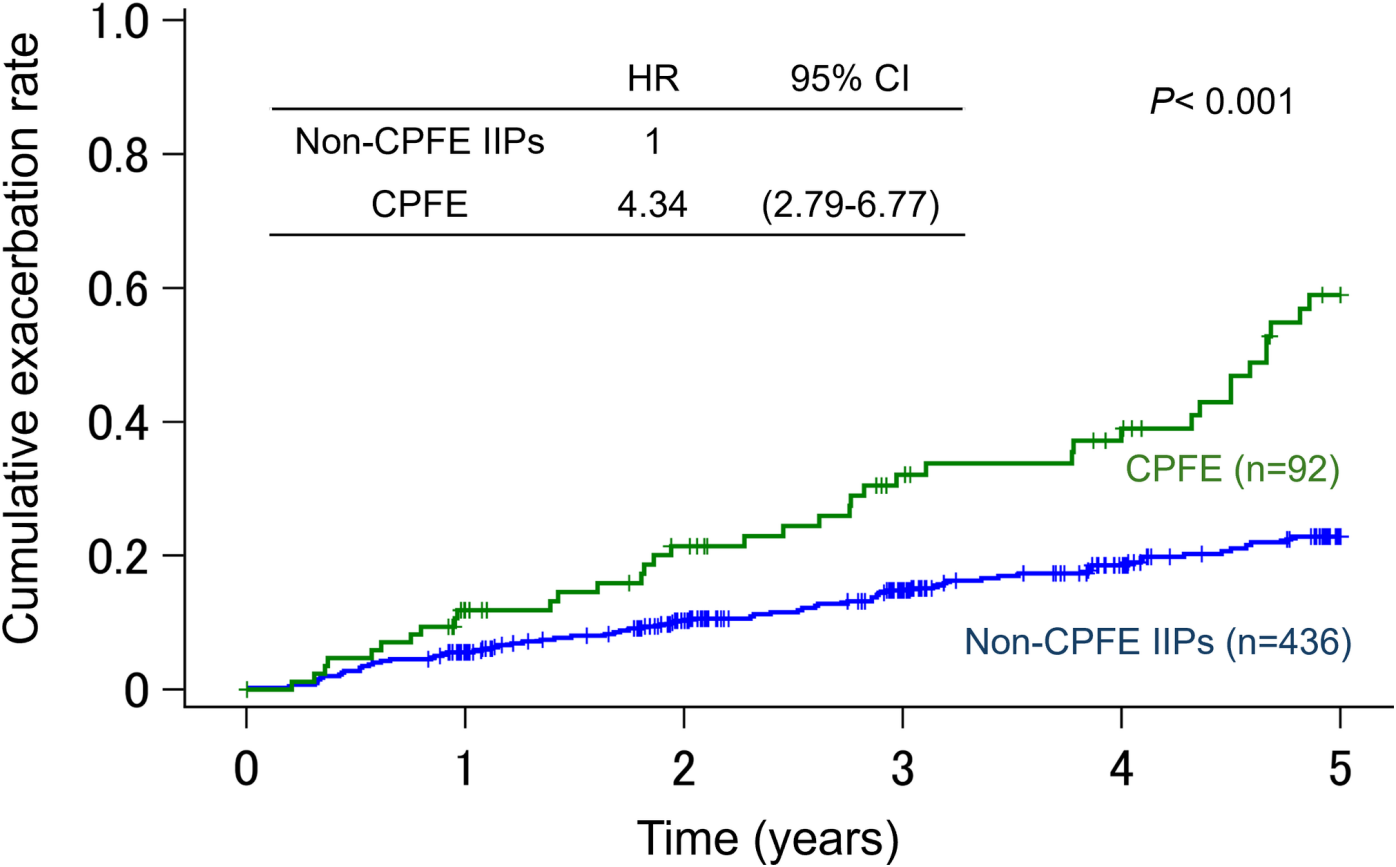
Kaplan–Meier plots for the cumulative incidence of acute exacerbation from the time of registration in patients with combined pulmonary fibrosis and emphysema (CPFE) or non‐CPFE idiopathic interstitial pneumonias (IIPs). CI, confidence interval; HR, hazard ratio. Multivariable analysis adjusted for age, sex, %FVC, and smoking pack‐years revealed that CPFE patients had a significantly higher risk of acute exacerbations compared with non‐CPFE IIP patients (HR of 4.34 [95% CI, 2.79–6.77], *p* < 0.001).

### 3.6 Predictors of Survival and Prognostic Relevance of IIP Subtype and CPFE Presence

Although the definition of CPFE does not specify the type of ILD according to the international classification criteria, prognosis may depend on whether the ILD is IPF or non‐IPF. To account for potential confounding by IIP subtype, we performed an additional analysis adjusted for IIP subtype, age, sex, %FVC, and pack‐year history. Even after adjustment for these factors including IIP subtype, CPFE patients still showed a significantly shorter overall survival compared with non‐CPFE IIP patients (HR of 1.85 [95% CI of 1.27–2.69], *p* < 0.001) (see Table S3).

Given that prognosis differs between IPF and non‐IPF IIPs, we performed a stratified analysis to evaluate whether the prognostic impact of CPFE varies according to the underlying IIP subtype. Patient characteristics stratified by the presence or absence of CPFE and further classified according to IIP subtype are presented in Table S4. Kaplan–Meier survival curves revealed significant differences among the four patient groups defined by CPFE status and IIP subtype (*p* < 0.001). CPFE consistently conferred worse survival within each IIP subtype, with CPFE/IPF patients showing the poorest outcomes among the four groups (Figure 4A). Correspondingly, an adjusted analysis revealed that CPFE/IPF patients had a higher mortality than did non‐CPFE/IPF patients (HR of 1.96 [95% CI, 1.23–3.13], *p* = 0.005) (Table S5). Similarly, CPFE/non‐IPF patients had a shorter overall survival than did non‐CPFE/non‐IPF patients, although this difference did not achieve statistical significance (HR of 1.67 [95% CI, 0.93–3.03], *p* = 0.086). Of note, the prognosis of CPFE/non‐IPF patients was similar to that of non‐CPFE/IPF patients (HR of 0.79 [95% CI, 0.47–1.33], *p* = 0.391). These findings indicate that, whereas CPFE consistently worsens prognosis regardless of underlying IIP subtype, the type of ILD further stratifies survival outcomes, suggesting that both factors should be considered together for optimal prognostic assessment. Consistent with these survival differences, the incidence of acute exacerbation was consistently higher in patients with CPFE (regardless of whether they had IPF or non‐IPF IIPs) compared with their non‐CPFE counterparts (Figure 4B and Table S6).

**FIGURE 4:**
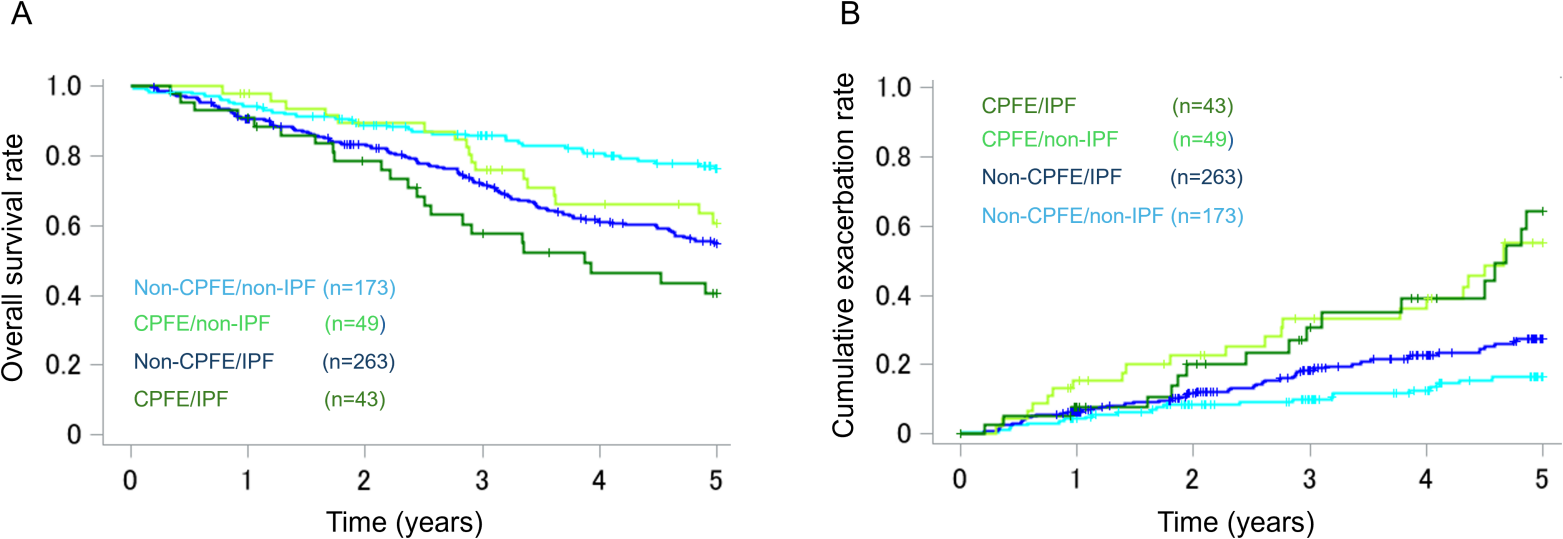
Kaplan–Meier curves of overall survival probability (A) and of the cumulative incidence of acute exacerbation (B) from the time of registration for IIP patients stratified by combined pulmonary fibrosis and emphysema (CPFE) status and underlying ILD subtype.

## 4 Discussion

Our large prospective cohort study validates the clinical utility of the internationally recommended CPFE classification based on visual assessment of CT scans and with a ≥ 5% emphysema threshold. This definition effectively identified a distinct patient subgroup with significantly worse survival outcomes compared with both non‐CPFE IIP and COPD patients, with the survival impact of CPFE being most pronounced in individuals with IPF. CPFE patients defined by this threshold showed several distinctive clinical characteristics, including markedly reduced overall survival, a higher rate of pulmonary hypertension–related death, and a pronounced decline in DLCO over time compared with non‐CPFE patients. Importantly, our cohort study revealed that individuals with CPFE had a significantly higher incidence of acute exacerbations compared with non‐CPFE IIP patients, representing critical prognostic information that extends beyond survival outcomes alone. This increased susceptibility to acute exacerbations was consistent across different IIP subtypes.

Our study demonstrates that visual assessment with six representative lung slices from apex to base provides a practical and effective method for quantification of emphysema extent in clinical practice [9]. Historically, CPFE studies have suffered from substantial diagnostic heterogeneity, with varied emphysema assessment methods and thresholds adopted across different investigations. This heterogeneity has posed challenges to comparisons of outcomes across studies and to establishment of standardized management approaches [3, 4, 5, 6, 7, 27]. Whereas the international statement defines a 5% threshold for research purposes and 15% for clinical classification [8], our findings show that visual assessment of emphysema at ≥ 5% effectively stratifies patients according to survival outcomes. The numerically lower survival observed with higher thresholds (10% and 15%) is consistent with the clinical utility of this approach, although a clear relationship between emphysema extent and survival was not firmly established. Beyond diagnostic accuracy, this approach also offers important practical advantages. Visual assessment requires no specialized software, can be performed during routine multidisciplinary discussions, and provides immediate results without computational delays. These findings support adoption of the 5% visual emphysema threshold as the standard diagnostic and prognostic tool in CPFE management.

Acute exacerbations represent critical prognostic events in ILDs, substantially affecting both survival outcomes and quality of life [28]. Although various frequencies of acute exacerbations in individuals with CPFE have been reported, previous investigations have been limited to single‐centre retrospective studies, making it difficult to determine the true incidence in the broader CPFE population [14, 15, 16, 17, 18]. Our large multicentre study is the first to demonstrate a significantly increased frequency of acute exacerbations in CPFE patients compared with non‐CPFE IIP patients in a prospective setting. The increased frequency of acute exacerbations in CPFE patients may be attributable to a heightened susceptibility to exacerbation triggers as a result of heterogeneous ventilation patterns caused by coexisting emphysema and fibrosis, as well as to persistent epithelial damage caused by chronic hypoxaemia associated with pulmonary hypertension [6, 29]. Consistent with our findings, acute exacerbations were previously found to represent a smaller proportion of causes of death in CPFE patients as a result of competing causes such as lung cancer and pulmonary hypertension [12]. However, another previous study reported that acute exacerbations in CPFE patients may have a worse prognosis than those in IPF patients [18], suggesting that acute exacerbations remain prognostic events as critical as other major complications in CPFE patients, and warranting enhanced surveillance and specialized management strategies. Furthermore, the lower proportion of acute exacerbation‐attributable mortality observed in our CPFE cohort compared with non‐CPFE IIP patients (10.0% vs. 17.8%) may reflect the higher burden of competing causes of death in CPFE, particularly lung cancer and pulmonary hypertension, which were both more prevalent in our CPFE group and may have reduced the relative proportion of acute exacerbation‐attributable deaths.

CPFE can occur in association with various forms of ILD, including IPF and non‐IPF conditions [19, 20, 21]. Whereas the international statement does not specify the underlying ILD type in the definition of CPFE, IPF and non‐IPF ILDs have distinct pathophysiologies and treatment responses [30]. We therefore evaluated whether the underlying IIP subtype might influence prognosis in CPFE patients. Our analysis revealed that prognostic outcomes varied substantially based on whether the fibrotic component was IPF or non‐IPF IIP. The most striking finding was that CPFE/non‐IPF IIP patients showed a prognosis similar to that of non‐CPFE/IPF patients, suggesting that emphysema significantly worsens the prognostic trajectory of non‐IPF IIP. Furthermore, CPFE/IPF patients manifested the worst survival outcomes among all groups. These findings indicate that both CPFE presence and ILD subtype should be considered together for optimal prognostic assessment. The similar prognosis apparent for CPFE/non‐IPF IIP and IPF alone also suggests that intensive management strategies similar to those adopted for IPF may benefit CPFE/non‐IPF IIP patients.

Our study has several limitations. First, it was conducted exclusively at Japanese institutions, which may limit the generalizability of our findings to other populations with different genetic backgrounds, environmental exposures, and health care systems. Validation with diverse ethnic populations is essential. Second, given the multiple centres with different CT scanners, variation in the quality of chest CT images may have affected the consistency of radiological assessments in our study. Third, treatment heterogeneity during the study period may have confounded outcomes. Fourth, our study focused exclusively on CPFE associated with IIPs, excluding CPFE patients with secondary ILD, which also may limit the generalizability of our findings to the broader CPFE population. Fifth, the enrollment period (2013–2016) preceded the widespread adoption of antifibrotic therapy, which may limit the generalizability of our findings to the current era of routine antifibrotic use. Although treatment heterogeneity may have influenced outcomes, sensitivity analyses adjusting for antifibrotic and immunosuppressive therapy confirmed that CPFE remained independently associated with worse survival across all models.

In conclusion, our large multicentre prospective cohort study has validated the clinical utility of visual CT assessment with a ≥ 5% emphysema threshold for CPFE diagnosis. CPFE patients showed significantly worse survival outcomes and higher acute exacerbation rates compared with non‐CPFE IIP patients. These findings support adoption of standardized CPFE classification criteria and emphasize the need for enhanced surveillance strategies and tailored management approaches for this high‐risk patient population.

## Author Contributions

**Katsuyuki Ichiki:** investigation, resources, writing – review and editing, data curation. **Kazuya Tsubouchi:** conceptualization, data curation, investigation, formal analysis, writing – original draft, writing – review and editing, methodology, resources, visualization. **Hiroaki Ogata:** conceptualization, investigation, methodology, writing – review and editing, data curation, resources. **Yasuaki Kotetsu:** conceptualization, investigation, writing – review and editing, methodology, data curation, resources. **Tomotsugu Takano:** conceptualization, investigation, methodology, writing – review and editing, data curation, resources. **Kazuhiro Yatera:** investigation, writing – review and editing, data curation, resources. **Kei Yamasaki:** data curation, resources, investigation, writing – review and editing. **Hiroshi Ishii:** data curation, resources, writing – review and editing, investigation. **Fumiaki Kiyomi:** visualization, writing – review and editing, formal analysis. **Kazunori Tobino:** data curation, resources, investigation, writing – review and editing. **Masaki Fujita:** data curation, resources, writing – review and editing, investigation. **Shohei Takata:** data curation, resources, investigation, writing – review and editing. **Tomohiro Ogawa:** investigation, writing – review and editing, resources, data curation. **Eiji Harada:** investigation, writing – review and editing, resources, data curation. **Masayuki Kawasaki:** investigation, writing – review and editing, resources, data curation. **Hiroshi Wataya:** investigation, writing – review and editing, data curation, resources. **Makoto Yoshida:** data curation, resources, writing – review and editing, investigation. **Masashi Komori:** investigation, writing – review and editing, resources, data curation. **Taishi Harada:** data curation, resources, writing – review and editing, investigation. **Isamu Okamoto:** supervision, project administration, writing – review and editing. **Yoichi Nakanishi:** conceptualization, methodology, supervision, funding acquisition, writing – review and editing, project administration. **Yasuhiko Kitasato:** investigation, writing – review and editing, resources, data curation. **Noriaki Nakagaki:** data curation, resources, writing – review and editing, investigation. **Masaki Okamoto:** investigation, writing – review and editing, resources, data curation.

## Funding

This study was supported by a grant from the Ministry of Education, Culture, Sports, Science and Technology (MEXT), Japan, by Boehringer Ingelheim, and by a grant from the Ministry of Health, Labour and Welfare (MHLW), Japan.

## Disclosure

No generative AI tools were used in the preparation of this manuscript.

## Ethics Statement

The study protocol was planned in accordance with the guidelines for research ethics of Kyushu University and was approved by the Institutional Review Board and Research Ethics Committee of the university (approval nos. #25‐135 and #555‐04). All participants provided written informed consent.

## Conflicts of Interest

The authors have reported the following: K.T.: Nippon Boehringer Ingelheim Co.; H.O.: Takeda Pharmaceutical Company Limited.; S.T.: AstraZeneca KK., Nippon Boehringer Ingelheim Co., Novartis Pharma KK, Sanofi K.K., Bristol‐Meyers Squib KK, Teijin Pharma KK, KYORIN Pharmaceutical Co., GlaxoSmithKline K.K.; K.Y.: Nippon Boehringer Ingelheim Co. Ltd., Chugai Pharmaceutical Co., TAIHO Phamaceutical Co. Ltd., Eli Lilly Japan K.K., Shionogi & Co. Ltd., DAIICHI SANKYO COMPANY, LIMITED., Kyowa Kirin Co. Ltd., Sumitomo Pharma Co. Ltd., Insmed Incorporated., TOA EIYO LTD., ONO PHARMACEUTICAL CO. LTD., GlaxoSmithKline K.K., Linical Co. Ltd., FUJIFILM Toyama Chemical Co. Ltd., TEIJIN HEALTHCARE LIMITED., Sanofi K.K, KYORIN Pharmaceutical Co. Ltd., Novartis Pharma K.K., Asahi Kasei Pharma Corporation., AstraZeneca K.K., MSD K.K, Takeda Pharmaceutical Company Limited., Meiji Seika Pharma Co. Ltd.; K. Tobino: Nippon Boehringer Ingelheim Co. GlaxoSmithKline K.K., AstraZeneca K.K.; E.H.: Nippon Boehringer Ingelheim Co.; I.O.: Daiichi Sankyo Company, Limited., CHUGAI PHARMACEUTICAL CO. LTD. ONO PHARMACEUTICAL CO. LTD. TAIHO PHARMACEUTICAL CO. LTD. Nippon Boehringer Ingelheim Co. Ltd., Bristol Myers Squibb K.K, AstraZeneca K.K., MSD Oncology K.K., Eli Lilly Japan K.K., Novartis Pharma K.K., Takeda Pharmaceutical Company Limited. K.Y. is an Editorial Board member of Respirology and a co‐author of this article. He was excluded from all editorial decision‐making related to the acceptance of this article for publication.

## Supporting information


**Table S1.** Treatment Regimens for Patients with Combined Pulmonary Fibrosis and Emphysema (CPFE), non‐CPFE Idiopathic Interstitial Pneumonias (IIPs), or Chronic Obstructive Pulmonary Disease (COPD) at Registration.
**Table S2.** Hazard Ratio (HR) for Mortality in Combined Pulmonary Fibrosis and Emphysema (CPFE) Patients Versus non‐CPFE Idiopathic Interstitial Pneumonia (IIP) Patients with Adjustment for Underlying Interstitial Lung Disease Subtype.
**Table S3.** Baseline Characteristics of Patients Stratified by Combined Pulmonary Fibrosis and Emphysema (CPFE) Status and Underlying Interstitial Lung Disease Subtype.
**Table S4.** Hazard Ratio (HR) for Mortality Stratified by Combined Pulmonary Fibrosis and Emphysema (CPFE) Status and Underlying Interstitial Lung Disease Subtype.
**Table S5.** Hazard Ratio for Acute Exacerbation Incidence Stratified by Combined Pulmonary Fibrosis and Emphysema (CPFE) Status and Underlying Interstitial Lung Disease Subtype.
**Figure S1.** Representative CT images at the aortic arch level illustrating emphysema extents of approximately 5%, 10%, 20%, and 30%, demonstrating the range of emphysema severity used as reference standards for visual assessment in this study.
**Figure S2.** Kaplan–Meier curves of overall survival probability from the time of registration for patients with combined pulmonary fibrosis and emphysema (CPFE), non‐CPFE idiopathic interstitial pneumonias (IIPs), or chronic obstructive pulmonary disease (COPD) according to emphysema thresholds of 10% (*A*) or 15% (*B*) for the definition of CPFE. CI, confidence interval.
**Figure S3.** Annual changes in %FVC (A) and %DLCO (B) from baseline over 5 years of follow‐up for patients with combined pulmonary fibrosis and emphysema (CPFE), non‐CPFE idiopathic interstitial pneumonias (IIPs), or chronic obstructive pulmonary disease (COPD). Data are medians ± the interquartile range. %DLCO, diffusion capacity for carbon monoxide (% predicted); %FVC, forced vital capacity (% predicted).

## Data Availability

The data that support the findings of this study are available on request from the corresponding author. The data are not publicly available due to privacy or ethical restrictions.
