# Supplementary material for: Clinical Outcomes and Acute Exacerbation Incidence in Combined Pulmonary Fibrosis and Emphysema
Source: Respirology. 2026 Jul 8;31(11):1117–25. doi: 10.1002/resp.70285 (PMC13728530; doi:10.1002/resp.70285)
Supplement: Supplementary file 1 [file RESP-31-1117-s001.docx]

**Supplementary Table S1.** Treatment Regimens for Patients with Combined Pulmonary Fibrosis and Emphysema (CPFE), non-CPFE Idiopathic Interstitial Pneumonias (IIPs), or Chronic Obstructive Pulmonary Disease (COPD) at Registration

|  | Non-CPFE IIPs  (*n* = 436) | CPFE  (*n* = 92) | COPD  (*n* = 441) | *P* value among all three groups | *P* value for non-CPFE IIPs vs. CPFE | *P* value for COPD vs. CPFE |
| --- | --- | --- | --- | --- | --- | --- |
| HOT | 41 (9.4) | 19 (20.7) | 70 (15.9) | 0.002 | 0.002 | 0.264 |
| ICS | 1 (0.2) | 4 (4.3) | 28 (6.3) | <0.001 | 0.004 | 0.630 |
| LABA | 14 (3.2) | 16 (17.4) | 93 (21.1) | <0.001 | <0.001 | 0.424 |
| ICS/LABA | 23 (5.3) | 22 (23.9) | 200 (45.4) | <0.001 | <0.001 | <0.001 |
| LAMA | 25 (5.7) | 37 (40.2) | 257 (58.3) | <0.001 | <0.001 | 0.002 |
| LABA/LAMA | 5 (1.1) | 9 (9.8) | 42 (9.5) | <0.001 | <0.001 | 1.000 |
| Theophylline | 3 (0.7) | 10 (10.9) | 88 (20.0) | <0.001 | <0.001 | 0.039 |
| Macrolide | 12 (2.8) | 11 (12.0) | 34 (7.7) | <0.001 | <0.001 | 0.183 |
| LTRA | 9 (2.1) | 2 (2.2) | 48 (10.9) | <0.001 | 1.000 | 0.006 |
| Steroid | 80 (18.3) | 9 (9.8) | 13 (2.9) | <0.001 | 0.046 | 0.003 |
| Immunosuppressant | 27 (6.2) | 1 (1.1) | 5 (1.1) | <0.001 | 0.068 | 1.000 |
| Pirfenidone | 50 (11.5) | 6 (6.5) | 0 (0.0) | <0.001 | 0.194 | <0.001 |
| Nintedanib | 1 (0.3) | 1 (1.4) | 0 (0.0) | 0.091 | 0.326 | 0.167 |

Data are presented as *n* (%). The *P* values were calculated with the chi-square test or Fisher’s exact test.

Abbreviations: HOT, home oxygen therapy; ICS, inhaled corticosteroids; LABA, long-acting β2-agonist; LAMA, long-acting muscarinic antagonist; LTRA, leukotriene receptor antagonist.

**Supplementary Table S2.** Hazard Ratio (HR) for Mortality in Combined Pulmonary Fibrosis and Emphysema (CPFE) Patients Versus non-CPFE Idiopathic Interstitial Pneumonia (IIP) Patients with Adjustment for Underlying Interstitial Lung Disease Subtype

|  | HR  (95% CI) | *P* value |
| --- | --- | --- |
| Non-CPFE IIP | 1 |  |
| CPFE | 1.85  (1.27–2.69) | <0.001 |

The HR and its 95% confidence interval (CI) were calculated with Cox proportional hazards regression models adjusted for age, sex, forced vital capacity (% predicted), pack-years of smoking, and IIP subtype.

**Supplementary Table S3.** Baseline Characteristics of Patients Stratified by Combined Pulmonary Fibrosis and Emphysema (CPFE) Status and Underlying Interstitial Lung Disease Subtype

|  | Non-CPFE/non-IPF  (*n* = 173) | Non-CPFE/IPF  (*n* = 263) | CPFE/non-IPF  (*n* = 49) | CPFE/IPF  (*n* = 43) |
| --- | --- | --- | --- | --- |
| Age (years) | 71 (65–78) | 73 (68–79) | 75 (68–80) | 73 (69–76) |
| Sex |  |  |  |  |
| Male | 105 (60.7) | 198 (75.3) | 45 (91.8) | 42 (97.7) |
| Female | 68 (39.3) | 65 (24.7) | 4 (8.2) | 1 (2.3) |
| Body mass index (kg/m^2^) | 23.4 (21.1–25.9) | 23.2 (21.1–25.4) | 22.6 (20.8–23.7) | 23.1 (21.2–24.2) |
| Medical history |  |  |  |  |
| Lung cancer | 2 (1.2) | 1 (0.4) | 3 (6.1) | 3 (7.0) |
| Other cancer | 18 (10.4) | 20 (7.6) | 7 (14.3) | 6 (14.0) |
| Smoking (pack-years) | 7.8 (0.0–41.5) | 23.0 (0.0–45.0) | 47.0 (30.0–75.0) | 56.0 (40.0–80.0) |
| Never | 77 (44.5) | 76 (28.9) | 4 (8.2) | 0 (0.0) |
| Former | 78 (45.1) | 158 (60.1) | 40 (81.6) | 38 (88.4) |
| Current | 18 (10.4) | 29 (11.0) | 5 (10.2) | 5 (11.6) |
| FVC (L) | 2.37 (1.83–2.98) | 2.45 (1.96–3.05) | 2.81 (2.19–3.28) | 3.04 (2.67–3.43) |
| FVC (% predicted) | 80.3 (68.0–94.3) | 82.3 (67.5–96.7) | 91.8 (74.9–103.0) | 92.0 (81.9–110.9) |
| FEV1 (L) | 1.87 (1.46–2.29) | 2.08 (1.59–2.44) | 2.01 (1.28–2.43) | 2.26 (2.02–2.58) |
| FEV1 (% predicted) | 81.1 (69.7–94.4) | 82.5 (74.1–94.7) | 73.4 (53.5–96.2) | 84.3 (69.6–96.8) |
| FEV1/FVC (%) | 81.2 (75.3–87.2) | 83.9 (77.9–88.6) | 67.1 (54.7–76.1) | 76.7 (66.9–82.8) |
| DLCO (% predicted) | 67.9 (57.6–81.2) | 62.1 (48.1–80.8) | 53.7 (51.2–60.6) | 46.6 (21.5–71.0) |
| KL-6 (U/mL) | 727 (465–1296) | 759 (500–1169) | 590 (347–866) | 719 (550–1124) |

Data are presented as median (interquartile range) for continuous variables and *n* (%) for categorical variables.

Abbreviations: DLCO, diffusion capacity of the lung for carbon monoxide; FEV1, forced expiratory volume in 1 s; FVC, forced vital capacity; IPF, idiopathic pulmonary fibrosis; KL-6, Krebs von den Lungen–6.

**Supplementary Table S4.** Hazard Ratio (HR) for Mortality Stratified by Combined Pulmonary Fibrosis and Emphysema (CPFE) Status and Underlying Interstitial Lung Disease Subtype

|  |  | Reference | | | |
| --- | --- | --- | --- | --- | --- |
|  |  | CPFE | | Non-CPFE | |
|  |  | IPF | Non-IPF | IPF | Non-IPF |
| CPFE | IPF |  | HR 2.44 (1.30–4.55)  *P* < 0.001 | HR 1.96 (1.23–3.13)  *P* = 0.005 | HR 4.17 (2.38–7.14)  *P* < 0.001 |
|  | Non-IPF |  |  | HR 0.79 (0.47–1.33)  *P* = 0.391 | HR 1.67 (0.93–3.03)  *P* = 0.086 |
| Non-CPFE | IPF |  |  |  | HR 2.08 (1.45–3.03)  *P* < 0.001 |
|  | Non-IPF |  |  |  |  |

The HR and its 95% confidence interval were calculated with Cox proportional hazards regression models adjusted for age, sex, forced vital capacity (% predicted), and pack-years of smoking.

Abbreviations: IPF, idiopathic pulmonary fibrosis.

**Supplementary Table S5.** Hazard Ratio for Acute Exacerbation Incidence Stratified by Combined Pulmonary Fibrosis and Emphysema (CPFE) Status and Underlying Interstitial Lung Disease Subtype

|  |  | Reference | | | |
| --- | --- | --- | --- | --- | --- |
|  |  | CPFE | | Non-CPFE | |
|  |  | IPF | Non-IPF | IPF | Non-IPF |
| CPFE | IPF |  | HR 1.52 (0.79–2.99)  *P* = 0.21 | HR 4.00 (2.27–7.14)  *P* < 0.001 | HR 8.33 (4.17–16.67)  *P* < 0.001 |
|  | Non-IPF |  |  | HR 2.63 (1.51–4.35)  *P* < 0.001 | HR 5.26 (2.94–10.00)  *P* < 0.001 |
| Non-CPFE | IPF |  |  |  | HR 2.04 (1.22–3.33)  *P* = 0.006 |
|  | Non-IPF |  |  |  |  |

The HR and its 95% confidence interval were calculated with Cox proportional hazards regression models adjusted for age, sex, forced vital capacity (% predicted), and pack-years of smoking.

Abbreviations: IPF, idiopathic pulmonary fibrosis.

**SUPPLEMENTARY FIGURE S1.**

Representative CT images at the aortic arch level illustrating emphysema extents of approximately 5%, 10%, 20%, and 30%, demonstrating the range of emphysema severity used as reference standards for visual assessment in this study.

**SUPPLEMENTARY FIGURE S2.** Kaplan-Meier curves of overall survival probability from the time of registration for patients with combined pulmonary fibrosis and emphysema (CPFE), non-CPFE idiopathic interstitial pneumonias (IIPs), or chronic obstructive pulmonary disease (COPD) according to emphysema thresholds of 10% (*A*) or 15% (*B*) for the definition of CPFE. CI, confidence interval.

**SUPPLEMENTARY FIGURE S3.** Annual changes in %FVC (*A*) and %DLCO (*B*) from baseline over 5 years of follow-up for patients with combined pulmonary fibrosis and emphysema (CPFE), non-CPFE idiopathic interstitial pneumonias (IIPs), or chronic obstructive pulmonary disease (COPD). Data are medians ± the interquartile range. %DLCO, diffusion capacity for carbon monoxide (% predicted); %FVC, forced vital capacity (% predicted).
